# Autochthonous *Dirofilaria repens* in Austria

**DOI:** 10.1186/1756-3305-7-226

**Published:** 2014-05-14

**Authors:** Katja Silbermayr, Barbara Eigner, Anja Joachim, Georg G Duscher, Bernhard Seidel, Franz Allerberger, Alexander Indra, Peter Hufnagl, Hans-Peter Fuehrer

**Affiliations:** 1 Institute of Parasitology, Department of Pathobiology, University of Veterinary Medicine Vienna, Veterinaerplatz 1, 1210 Vienna, Austria; 2 Technical Office of Ecology and Landscape Assessment, Persenbeug, Austria; 3 Austrian Agency for Health and Food Safety, Division of Public Health, Vienna, Austria

**Keywords:** Mosquito, Dirofilariosis, *Anopheles maculipennis*, *Anopheles algeriensis*

## Abstract

**Background:**

In Europe animal and human infections due to *Dirofilaria repens* are increasing.

**Findings:**

In a nationwide screening for filarioid parasites in Austria, 7,632 mosquitoes were collected from June till October 2012 and divided into 437 pools according to same trapping date and sight and mosquito species. For the molecular detection, a real-time PCR approach was followed by conventional PCR. *D. repens* was detected in the villages Moerbisch and Rust, Burgenland in one *Anopheles maculipennis* group and one *Anopheles algeriensis* species pool, respectively.

**Conclusions:**

The geographical distribution of the two positive pools points to the invasion of *D. repens* from Eastern neighboring countries. The finding of *D. repens* in mosquito vectors suggests the occurrence of the causative agent for cutaneous dirofilariosis in Austria.

## Background

In the past few years a new emergence of filarioid diseases like dirofilariosis has been noted, particularly in Europe where the human infections due to *Dirofilaria (D.) repens* are increasing [1]. Although several *Dirofilaria* species are considered zoonotic, *D. repens* is the most common species causing human dirofilariosis in temperate areas [1,2]. Therefore, infections with *D. repens* are of growing concern for veterinary and public health. In Germany, *D. repens*[3] and more recently *D. immitis*[4] has been detected inside their mosquito vectors. The higher the parasite prevalence in the mosquito host, the greater the risk of transmission to mammalian hosts [5]. High frequencies are reported in humans from regions with high prevalence of *D. repens* in animal hosts which are inhabited by zooanthropophilic (so-called bridge) vectors [6]. Of the 1,410 subcutaneous/ocular human dirofilariosis cases so far reported worldwide, 16 were described in Austria from 2000 – 2011 [1]. The first suspected autochthonous case of dirofilariosis in an Austrian patient without recent travel history was reported in 2008 [7]. In the following year *D. repens* positive blood samples were detected in dogs from the Eastern Austrian province Burgenland and the district of Gaenserndorf (Lower Austria). Simultaneous screening of mosquito pools from the same species and the same trapping area with real-time PCR (qPCR), however, did not detect *D. repens* in the vector [8]. Since humans are at risk of infection wherever canine dirofilariosis is present [5] a nationwide screening for mosquito-borne filarioid parasites was initiated. Until the year 2012, the autochthonous presence of this emerging filarioid disease in Austria was not confirmed since no *D. repens* was detected in their mosquito vectors. Here, the first autochthonous case of *D. repens* detected in Austrian mosquitoes is described, emphasizing the possible transmission to humans in areas by infected vectors.

## Methods

**Figure 1:**
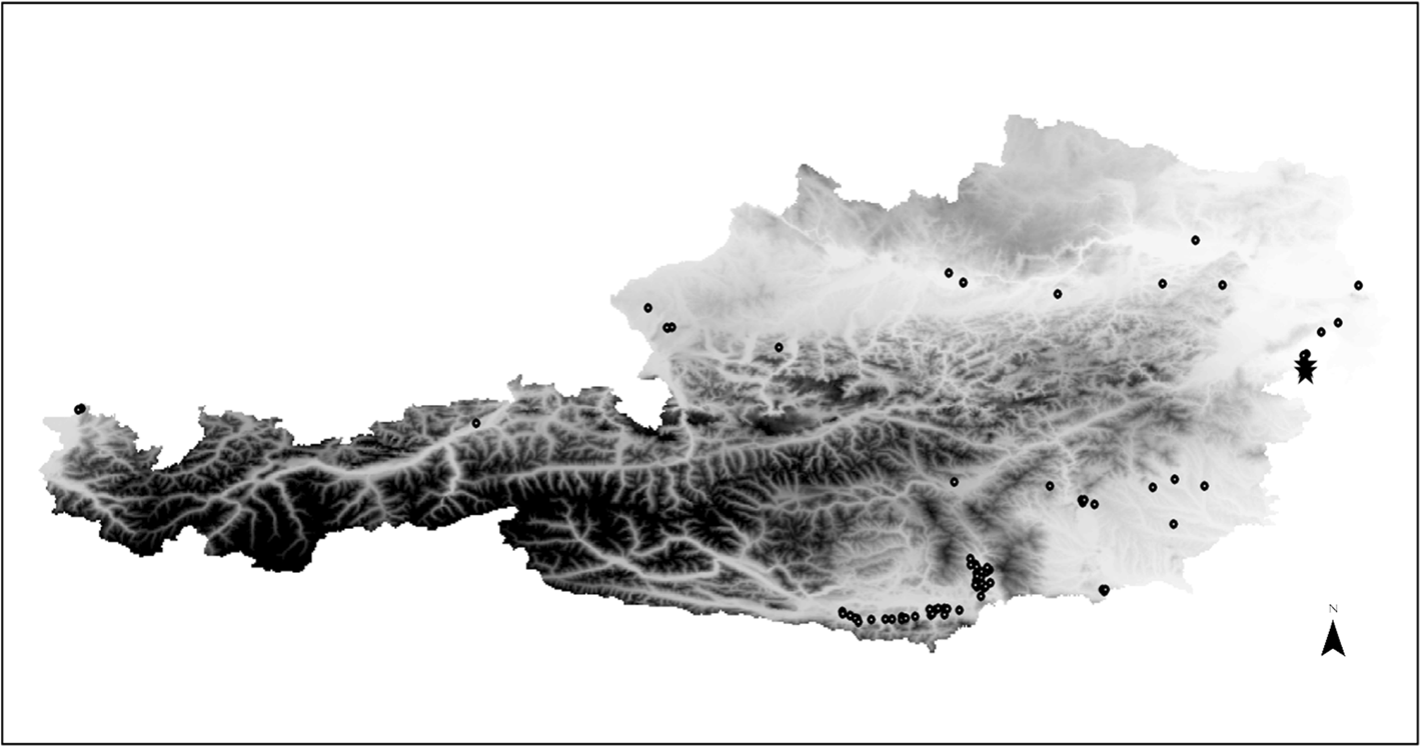
**Map of sampling locations and sites of *D. repens *detection.** Trapping sites (◦) and site of *D. repens* positive pools (★) in Rust and Moerbisch.

Mosquitoes were collected using BG sentinel™ traps (Biogents AG, Regensburg, Germany) from June till October 2012 in a nationwide mosquito monitoring and surveillance program (Figure 1). After collection the mosquitoes were transported alive to the laboratory and stored at −80°C until species differentiation. Morphometric evaluation was performed microscopically and specimens from the same site, trapping date and species were pooled. The 7,632 collected mosquitoes were divided into 437 pools according to the species, trapping-site and trapping-date, with an average pool size of 17 individuals. One 3 mm Tungsten Carbide Bead (Qiagen, Hilden, Germany) was added to each mosquito pool; after homogenization in a TissueLyser II (Qiagen, Hilden, Germany), DNA extraction was performed with a DNA Isolation Kit for Cells and Tissues (Roche, Basel, Switzerland). For the molecular detection of *Dirofilaria* spp. a qPCR approach [9] was applied to screen the mosquito pools for filarioid DNA. The following primers and a fluorescent labelled probe were used. FILA-F (5’ TGG ATT AGT ACC CAG GTA ATC 3’) and FILA-R (5’ CCA AAG AAA AAT CTA AAG TCA GTC 3’) and probe FILA-P (5’ HEX AAC AAA ACT TTA CTC CCGA-BHQ1 3’). The qPCR positive pools were subsequently examined by conventional PCR [10] for sequencing and species discrimination. The PCR product was subjected to Illustra™ ExoStar™1-Step (GE Healthcare, Buckinghamshire, United Kingdom) for subsequent DNA-Sanger-Sequencing by Microsynth (Microsynth AG, Balgach, Switzerland). After alignment of the forward and reverse sequence, a consensus sequence was constructed and compared to GenBank® entries using BLAST (http://blast.ncbi.nlm.nih.gov).

## Findings

Two pools tested positive in the qPCR assay and were subjected to conventional PCR for the amplification and subsequent sequencing of a 667-bp long fragment spanning the cytochrome oxidase subunit I (COI) gene. The first positive pool collected on September 13th, 2012 originated from the village of Moerbisch, Burgenland. All 18 individuals of the pool belonged to the *Anopheles maculipennis* (Meigen, 1880) group. The mosquitoes of the second positive pool were caught in the village of Rust, Burgenland on September 14th, 2012 and consisted of 14 individuals, all belonging to the *Anopheles algeriensis* (Theobald, 1903) species. Both samples revealed a 100% similarity to a *D. repens* entry in GenBank® (*D. repens* COI gene: JF461458.1).

## Conclusions

The geographical distribution of the two positive pools points to the possible invasion of *D. repens* from Eastern neighboring countries (Figure 1). The locations of recent cases in humans [7] and dogs [8] overlap in districts close to Hungary [11]. The first Austrian patient – supposedly without any travel history – even worked as border patrol at the Austrian-Hungarian border, approximately 50 km from the positive trapping site at Rust. However, the presence of the nematode DNA does not provide full evidence of the autochthonous biological cycle in Austria and further investigations on the vectorial role of indigenous mosquitoes and their vector competence are needed. Up until now autochthonous occurrence of *D. repens* has never been shown for Austria. The finding of *D. repens* in mosquito vectors suggests the occurrence of the causative agent for cutaneous dirofilariosis in the Eastern region of Austria.

## Competing interests

Authors declare no conflict of interest and no competing interests related to this article.

## Authors’ contributions

KS has performed the molecular biological lab work (including sequence alignment) and compiled the results for the manuscript. BE performed the technical assistance in the molecular biological work (PCRs and sequencing), AJ realized and coordinated the research project at the institute and contributed to the writing of the manuscript, GGD assisted in the computational analysis with GIS, BS collected and differentiated the specimen, FA initiated and designed the research project, AI supervised the laboratory work and edited the manuscript, PH did the molecular biological work (Sample preparation and DNA extraction), HPF provided scientific and technical assistance and was responsible for the overall implementation of the research project. All authors read and approved the final manuscript.

## Authors’ information

Katja Silbermayr holds a PhD in veterinary medicine and currently works as postdoc at the Institute of Parasitology, Vetmeduni Vienna, Austria. She is a 3rd-year resident of the European Veterinary Parasitology College with a strong research focus on vector-borne- and parasitic-skin-diseases.
